# Proteomic and Phosphoproteomic Profiling Reveals the Oncogenic Role of Protein Kinase D Family Kinases in Cholangiocarcinoma

**DOI:** 10.3390/cells11193088

**Published:** 2022-09-30

**Authors:** Yun Lu, Xiangyu Li, Kai Zhao, Yuanxin Shi, Zhengdong Deng, Wei Yao, Jianming Wang

**Affiliations:** 1 Department of Biliary and Pancreatic Surgery/Cancer Research Center Affiliated Tongji Hospital, Tongji Medical College, Huazhong University of Science and Technology, Wuhan 430030, China; 2 Department of Oncology Affiliated Tongji Hospital, Tongji Medical College, Huazhong University of Science and Technology, Wuhan 430030, China; 3 Affiliated Tianyou Hospital, University of Science & Technology, Wuhan 430064, China

**Keywords:** cholangiocarcinoma, phosphoproteomics, protein kinase, protein kinase D

## Abstract

Cholangiocarcinoma (CCA) is a lethal malignancy in the hepatobiliary system, with dysregulated protein expression and phosphorylation signaling. However, the protein and phosphorylation signatures of CCAs are little-known. Here, we performed the proteomic and phosphoproteomic profiling of tumors and normal adjacent tissues (NATs) from patients with CCA and predicted eleven PKs high-potentially related to CCA with a comprehensive inference of the functional protein kinases (PKs) (CifPK) pipeline. Besides the two known CCA-associated PKs, we screened the remaining candidates and uncovered five PKs as novel regulators in CCA. Specifically, the protein kinase D (PKD) family members, including PRKD1, PRKD2, and PRKD3, were identified as critical regulators in CCA. Moreover, the pan-inhibitor of the PKD family, 1-naphthyl PP1 (1-NA-PP1), was validated as a potent agent for inhibiting the proliferation, migration, and invasion ability of CCA cells. This study reveals new PKs associated with CCA and suggests PRKD kinases as novel treatment targets for CCA.

## 1. Introduction

Cholangiocarcinoma (CCA), accounting for about 15% of primary liver cancer, is the second most prevalent cancer after hepatocellular carcinoma (HCC) [1]. CCA is a highly lethal malignancy in the biliary tree, and its incidence and mortality continue to increase [2,3]. Currently, the prognosis of CCA is poor, with a 5-year overall survival (OS) rate of 7–20% [1,2]. The causal mechanism is complex, with prevalent mutations, such as kinase signaling [4,5] and fibroblast growth factor receptor 2 (FGFR2) fusions [6,7,8,9]. Moreover, the molecular pathogenesis of CCA includes aberrant signaling pathways, such as receptor tyrosine kinase (RTK) [10], RAS-mitogen-activated protein kinase (MAPK) [11], and phosphoinositide 3-kinase (PI3K)/protein kinase B (AKT)/mammalian target of rapamycin (mTOR) signaling [12]. Protein kinases (PKs) are central regulators for activating or inhibiting these signaling pathways. In 2021, Infigratinib, a derivative of anilino-pyrimidine, was approved by the Food and Drug Administration (FDA) for treating CCAs with FGFR2 fusion [13]. In addition, several inhibitors for designated PKs, such as ROS tyrosine kinase fusion [14] and serine/threonine-protein kinase B-RAF (BRAF) [15], have been applied to evaluate their efficacy in early clinical trials of CCA with a small sample size. In this regard, identifying dysregulated signaling and aberrant PKs could facilitate the early diagnosis and discovery of effective therapeutic drugs for CCAs.

Recently, liquid chromatography with tandem mass spectrometry (LC-MS/MS) techniques has greatly advanced the identification of molecular alterations and regulations in CCA [16,17]. LC/MS-MS phosphoproteomics is a branch of proteomics focusing on identifying phosphorylation events, which has enabled the quantification of over 10,000 phosphorylation sites per experiment. Given the enormous complexity of phosphorylated proteomics data, several bioinformatics tools have been applied to analyze phosphorylation data, including NetworKIN [18], iGPS [19], and KESA [20]. These tools predicted kinase-specific substrates based on clustering and enrichment methods, which can significantly benefit discovery of new PK targets for tumor treatment.

In order to investigate the dysregulated phosphorylation events and uncover the potential PKs for CCA, we profiled proteomes and phosphoproteomes for four pairs of poorly differentiated tumors and corresponding NATs from CCA patients by using the LC-MS/MS combined with tandem mass tag (TMT) labeling. We quantified 7667 proteins and 12,027 phosphorylation sites (p-sites) of 4088 phosphoproteins from proteomic and phosphoproteomic data. In addition, we predicted 11 highly potential PKs and validated five newly CCA-associated PKs by the comprehensive inference of the functional PKs (CifPK) pipeline, mainly determined by the KESA and iGPS analysis. Specifically, three PK members of the protein kinase D (PKD) family, PRKD1, PRKD2, and PRKD3, were experimentally validated as oncogenic roles in CCA with the small interfering RNAs (siRNAs) and a selective inhibitor, 1-naphthyl PP1 (1-NA-PP1). Altogether, our study profiled the landscapes of protein expression and phosphorylation modification in CCA, which provided new insights into the functional PKs and treatment targets for CCA.

## 2. Materials and Methods

### 2.1. Collection of Clinical Samples and Information

Four patients with ECCA without chemotherapy or radiotherapy were enrolled in this study. Patients independently signed an informed consent form, and agreed to donate tumor tissue and matched NAT tissue for scientific research. The demographic characteristics and clinical information of the patients in this study are presented in Table S1. The tumor and NAT tissues were collected immediately after surgery. All the samples were dipped into tissue storage solution (Miltenyi Biotec, Cologne, Germany,) and transported on ice.

### 2.2. Extraction and Digestion of Protein

The samples were lysed by sonification in lysis buffer (1% Triton X-100, 1% protease inhibitor, and 1% phosphorylase inhibitor). After the centrifugation at 20,000× *g*, 4 °C for 10 min, the supernatants were collected to measure the protein concentration with the BCA kit (Beyotime, Shanghai, China). For the digestion of proteins, the equal protein solution was treated with 1: 50 trypsin-to-protein for overnight treatment. Then, the protein solution was treated with 5 mm DL-dithiothreitol (DTT, Sigma-Aldrich, St. Louis, America) at 56 °C for 30 min, followed by 11 mm iodoacetamide (IAM, Sigma-Aldrich, St. Louis, America) at room temperature in the dark for 15 min.

### 2.3. TMT Labelling

The tryptic peptides were firstly dissolved in 0.5 M TEAB. Each channel of the peptide was labeled with its respective TMT reagent (based on the manufacturer’s protocol, Thermo Scientific, Massachusetts, America) and incubated for 2 h at room temperature. Five microliters of each sample were pooled, desalted, and analyzed by MS to check labeling efficiency. After the labeling efficiency check, samples were quenched by adding 5% hydroxylamine. The pooled samples were then desalted with Strata X SPE column (Phenomenex) and dried by vacuum centrifugation. The TMT labeling data was listed in Table S2.

### 2.4. HPLC Fractionation

The sample was fractionated by high pH reverse-phase HPLC using Agilent 300 Extend C18 column (5 μm particles, 4.6 mm ID, 250 mm length). Briefly, peptides were separated with a gradient of 2% to 60% acetonitrile in 10 mm ammonium bicarbonate pH 10 over 80 min into 80 fractions. Then, the peptides for proteomic/phosphoproteomics were combined into 12/6 fractions and dried by vacuum centrifuging.

### 2.5. Enrichment of Phosphopeptides

The peptide mixtures were first incubated with IMAC microspheres suspension with vibration in a loading buffer (50% acetonitrile/0.5% acetic acid). To remove the non-specifically adsorbed peptides, the IMAC microspheres were washed with 50% acetonitrile/0.5% acetic acid and 30% acetonitrile/0.1% trifluoroacetic acid, sequentially. To elute the enriched phosphopeptides, the elution buffer containing 10% NH4OH was added, and the enriched phosphopeptides were eluted with vibration. The supernatant containing phosphopeptides was collected and lyophilized for LC-MS/MS analysis.

### 2.6. LC-MS/MS Analysis

The peptides were dissolved in liquid chromatography mobile phase A and separated using an EASY-NLC 1200 ultra-high-performance liquid phase system. Mobile phase A was an aqueous solution containing 0.1% formic acid and 2% acetonitrile; mobile phase B was an aqueous solution containing 0.1% formic acid and 90% acetonitrile. The gradient settings were: 0–38 min, 6%–22% B; 38–52 min, 22%–32% B; 52–56 min, 32%–80% B; 56–60 min, 80% B. The peptides were separated by the UHPLC system and then injected into the NSI ion source for ionization before being analyzed by Q Exactive™ HF-X mass spectrometry. The ion source voltage was set at 2.1 kV, and the peptide parent ions and their secondary fragments were detected and analyzed using a high-resolution Orbitrap. The primary mass spectrometry scan range was set to 350–1400 *m*/*z* with a resolution of 60,000, while the secondary mass spectrometry scan range was set to a fixed starting point of 100 *m*/*z* with a resolution of 30,000. The pool was fragmented using a fragmentation energy of 28%, and the secondary mass spectrometry was performed in the same order. To improve the effective utilization of the mass spectra, the automatic gain control (AGC) was set to 5 × 10^4^, the signal threshold was set to 8.3 × 10^4^ ions/s, the maximum injection time was set to 60 ms, and the dynamic exclusion time of the tandem mass spectrometry scan was set to 15 s seconds to avoid repeated scanning of the parent ions. When the location probability of the particular phospho-site was >0.75, it was considered the secure site.

### 2.7. Standard Database Search

The MS/MS raw data were searched based on the MaxQuant (Version 1.6.15.0) [21]. The human reference proteome was downloaded from UniProt (Version 202012) [22], which contained 20,395 human protein sequences. The cleavage method was set as Trypsin/P, allowing up to 2 missing cleavages. The minimum length of peptides was set to 7, and the maximum of modifications in one peptide was set to 5. The fixed modification was set as Carbamidomethyl (C), while Oxidation (M) and Acetyl (Protein N-term) were the variable modifications for the searching of proteomic and phosphoproteomic data. Besides, the variable modification also included Pospho (STY) for searching phosphoproteomic data only. The false discovery rates (FDRs) for the peptide-spectrum match (PSM) and protein were set to <1%.

### 2.8. Proteomic Analysis

First, the PCA was performed for quality control (QC), based on quantifying all proteins from proteomic data. Then, the fold change of a protein in the tumor against the corresponding NAT was calculated to identify the differentially expressed proteins (DEPs)for each patient based on the reporter intensities of the tumor and NAT. The fold change cutoff for DEPs was set as ≥2 or ≤0.5.

### 2.9. Phosphoproteomic Analysis

To exclude the effect of protein expression in the quantification of phosphorylation, the quantified p-sites were normalized by dividing the quantification value of the corresponding protein, if available, for each sample. The normalized p-sites were used for further analysis and PCA-based QC. For each patient, the fold change of a p-site was computed based on the reporter intensities of the tumor and NAT. The factor (≥2 or ≤0.5-fold change) was adopted to identify differentially regulated p-sites (DRPs). The differentially phosphorylated proteins (DPPs) are defined as the phosphoproteins containing at least one DRP.

### 2.10. Public P-Site Resources

The experimentally identified p-sites of human phosphoproteins were downloaded and integrated from five public databases, including UniProt [22], EPSD [23], dbPTM 2019 [24], PhosphoSitePlus [25], and Phospho.ELM 9.0 [26]. There are 534,457 known p-sites in 30,637 human phosphoproteins, including 314,856 pS (58.91%), 149,113 pT (27.90%), and 70,488 pY (13.19%).

### 2.11. GO Functional Enrichment Analysis

Proteins were classified by GO annotation into three categories: biological process, cellular compartment and molecular function. For each category, a two-tailed Fisher’s exact test was employed to test the enrichment of the differentially expressed protein against all identified proteins. The GO with a corrected *p*-value < 0.05 is considered significant.

### 2.12. KEGG Functional Enrichment Analysis

The functional enrichment analysis of DEPs was computed by the hypergeometric test based on the hallmark gene sets. First, the hallmark annotations were downloaded from the Molecular Signatures Database (MSigDB, v7.2, https://www.gsea-msigdb.org/gsea/msigdb (accessed on 1 October 2021) [27], which contained 50 well-defined biological states or processes. Then, we defined and counted four statistics, as below:

*N* = number of identified proteins annotated with at least one gene set

*n* = number of DEPs annotated with at least one gene set

*M* = number of identified proteins annotated with the defined gene set *t*

*m* = number of DEPs annotated with the defined gene set *t*

Then, the enrichment ratio (*E ratio*) and the corresponding p-value were calculated with hypergeometric distribution, as below:

E ratio=mMnN

$$p=\{\begin{array}{c} \overset{n}{\underset{m^{\prime }=m}{\sum }}\frac{\left(\begin{array}{c} M\\ m^{\prime } \end{array}\right)\left(\begin{array}{c} N-M\\ n-m^{\prime } \end{array}\right)}{\left(\begin{array}{c} N\\ n \end{array}\right)},\;\left(E\;ratio\geq 1\right)\\ \overset{m}{\underset{m^{\prime }=0}{\sum }}\frac{\left(\begin{array}{c} M\\ m^{\prime } \end{array}\right)\left(\begin{array}{c} N-M\\ n-m^{\prime } \end{array}\right)}{\left(\begin{array}{c} N\\ n \end{array}\right)},\left(E\;ratio<1\right) \end{array}$$

Then, the hypergeometric test was also used for the MSigDB-based enrichment analysis of DPPs.

### 2.13. The CifPK Pipeline

The three main steps of the CifPK pipeline were enrichment-based analysis, KSEA-based prediction, and integrating results from the two methods.

In the enrichment-based analysis, the upstream PKs of normalized p-sites were first predicted by using the in vivo Group-based Prediction System (iGPS, http://igps.biocuckoo.org/ (accessed on 1 October 2021)) [19] with the default parameters (‘Low threshold’ and ‘Experiment/STRING PPI’). For each patient, the hypergeometric test was used to identify the significantly associated PKs with DRPs, based on the computationally site-specific kinase-substrate relations (ssKSRs). The *p* < 0.05 was adopted as the threshold, and the PKs statistically enriched in ≥2 patients were reserved for further analysis.

In the KSEA-based prediction, the R package KSEAapp (Version 0.99.0) [20] for Kinase-Substrate Enrichment Analysis (KSEA) [20,28] was used to identify CCA-associated PKs based on the fold change of normalized p-sites and the known kinase-substrate relations. The exact cutoff (*p* < 0.05) was set as the threshold, and the PKs with significantly changed kinase activity in ≥2 patients were output for further analysis.

The results obtained from enrichment- and KSEA-based methods were integrated and evaluated for the inference of functional PKs. Obviously, PKs in the intersection set of two methods have a higher probability of association with the CCA. Moreover, some PKs predicted by the two methods were related to overlapped PKs in the known function and classification of the kinase family. Therefore, these highly-likely potential PKs were considered CCA-Associated PKs.

### 2.14. Cell Culture and Transfection

TFK-1 and HuCCT1 cells were cultured in RPMI 1640 medium with 10% fetal bovine serum (FBS). All cells were cultured in a humidified incubator at 37 °C, 5% CO_2_. All siRNAs used in this study (Table S4) were obtained from RIBO Biotechnology (China) and transfected into CCA cells using Lipotransfectamine 3000 (Thermo Fisher Scientific, Waltham, MA, USA) according to the instructions. Briefly, CCA cells were seeded in a six-well plate until the cell density reached approximately 60%. 200 μL serum-free OPTI-MEM (Gibco) was mixed with 5 uL of Lipotransfectamine 3000 and 5 μL siRNA (20 μM), respectively, for 5 min at room temperature before mixing the above liquids, and the mixture was added dropwise to the six-well plate after 20 min at room temperature. The volume of transfection was then adjusted to 2 mL per well by adding the serum-free OPTI-MEM. Cells were transfected under serum-free OPTI-MEM conditions for 6–8 h before being replaced with a complete medium and incubated in the incubator for 24 h for subsequent experiments. Three siRNA were initially tried for silencing of each single PK, and the most efficient siRNA were selected for subsequent functional assays (Figure S4).

### 2.15. Western Blotting

Western blotting assay was performed as described previously [29]. The antibodies used in this study are as follows: β-actin antibody (PTM Bio, Hangzhou, China), PRKD1 (ABclonal, China, A2417), PRKD2 (ABclonal, China, A17670), PRKD3 (ABclonal, China, A7084), PRKCA (ABclonal, China, A11107), PRKCZ (ABclonal, China, A5714), CSNK1A1 (ABclonal, China, A9308), CSNK1A2 (ABclonal, China, A21368), PDK2 (ABclonal, China, A4012), BCKDK (ABclonal, China, A20909), Phospho-Scr-S17 (ABclonal, China, AP0522), and Phospho-β-catenin-S552 (ABclonal, China, AP0579).

### 2.16. Cell Proliferation and Drug Cytotoxic Assay In Vitro

Cell Counting Kit-8 assay (CCK-8) was performed to detect cell proliferation capacity. TFK-1 and HuCCT1 cells with a density of 2 × 10^3^ cells per well were seeded in 96-well plates. Each group had three replicates (*n* = 3). At the indicated time-points, 10 μL of CCK-8 solution was added to every well, and the cells were incubated at 37 °C and 5% CO_2_ for 2 h according to instruction. Then, we measured the absorbance at 450 nm at different time points with the plate reader (Bio-Tek Elx 800, Vermont, USA) to assess cell proliferation. All experiments were performed three times following the same procedure. To detect the half-maximal inhibitory concentration (IC50) of 1-NA-PP1 in CCA cells, TFK-1 and HuCCT1 cells were seeded in 96-well plates with 2 × 10^3^ cells per well and treated with 1-NA-PP1 at different concentrations for 48 h. Then other procedures were the same as the CCK-8 assay described above.

### 2.17. Wound Healing

The CCA cells for detection were inoculated in 6-well plates; when they grew to nearly 90% confluence, we scratched the wound vertically across the center of the well gently with the tip of a 200 μL pipe. After washing three times with PBS, the CCA cells were cultured in a serum-free medium at 37 °C, 5% CO_2_. Then, wound healing was observed and recorded with a microscope (Nikon, Tokyo, Japan) at 0 and 24 h, respectively. All experiments were repeated three times.

### 2.18. Transwell Assay

To examine cell migration capacity, we suspended 1 × 10^5^/mL CCA cells, which had been treated by starvation for 12 h before, and placed 5 × 10^4^ CCA cells per well with 200 μL serum-free medium in the upper Transwell chamber of a 24-well Transwell plate (8 μm pore size; Corning), and 500 μL of a complete medium in the lower chamber. After co-culturing for 24 h, the CCA cells on the sub-membrane surface were fixed with 4% paraformaldehyde and stained with the crystalline violet solution. The stained cells were then counted with a Nikon light microscope (Nikon, Japan). In the case of the invasion assay, 60 μL of Matrigel matrix gel was placed in the upper Transwell chamber (BD Biosciences, San Jose, NJ, USA). Other operations performed were the same as the cell migration assay described above. All experiments were carried out three times following the same procedure.

## 3. Results

### 3.1. The Procedure for Analysis of CCA-Associated PKs

**Figure 1:**
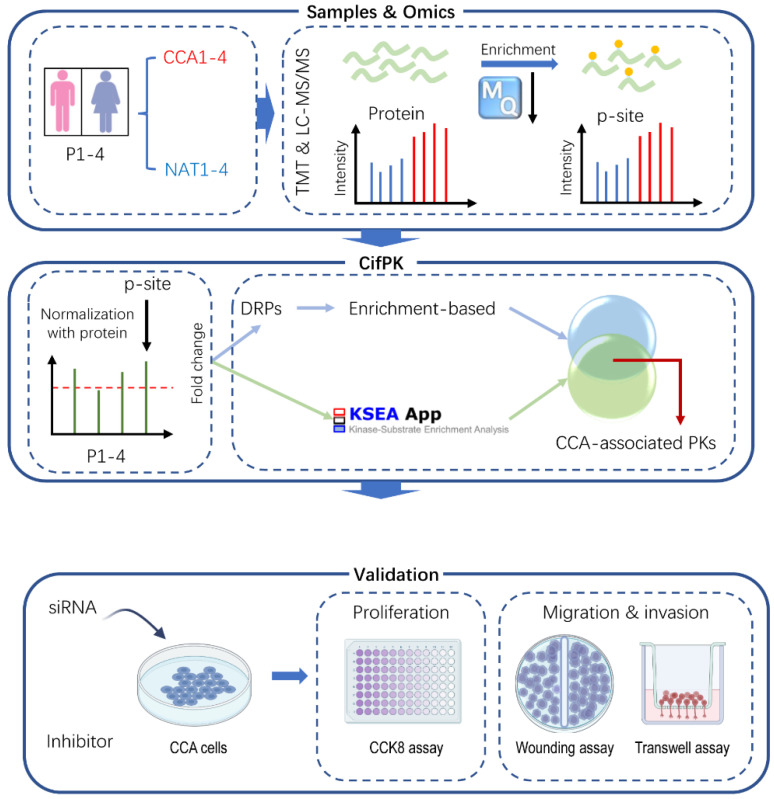
The workflow of this study. First, TMT-labeled and LC-MS/MS-based proteomics and phosphoproteomics of CCA tumors with corresponding NAT samples were profiled. Then, after the normalization of quantified p-sites with corresponding proteins, an integrated pipeline CifPK was developed to predict the CCA-associated PKs. Last, the experimental assays were performed to validate the effect of potential CCA-associated PKs by using siRNAs and selective inhibitors of PKs.

To systematically characterize the kinome of CCA, we first collected four poorly differentiated CCA tissues with corresponding NATs from Tongji Hospital (Figure 1); the detailed clinical features of these patients are shown in Table S1. Next, the proteomic and phosphoproteomic profiling of CCA tumors with NATs were performed by using LC-MS/MS (Figure 1). After the extraction and digestion of proteins from each sample, peptides were labeled with TMT reagent and fractionated, and phosphopeptides were enriched with immobilized metal ion affinity chromatography (IMAC).

Then, we systematically analyzed the proteomic and phosphoproteomic profiling, and developed an integrated pipeline, comprehensive inference of functional PKs (CifPK), to identify the potential PKs associated with CCAs by integrating the enrichment- and KSEA-based methods to predict CCA-associated PKs from phosphoproteomic data (Figure 1). To validate the functional roles of CCA-associated PKs, further experiments were performed in both TFK-1 and HuCCT1 by using specific siRNAs and selective inhibitors of PKs (Figure 1).

### 3.2. The Proteomic and Phosphoproteomic Profiling of CCAs

**Figure 2:**
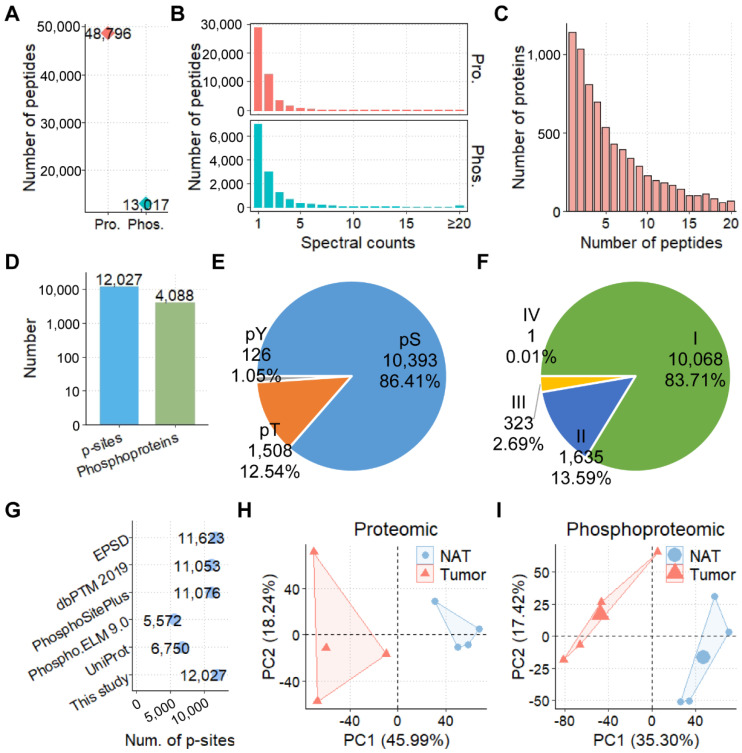
The proteomic and phosphoproteomic profiling of CCAs: (**A**) numbers of peptides and phosphopeptides identified in all samples; (**B**) distributions of MS/MS spectral counts of peptides and phosphopeptides obtained from proteomics and phosphoproteomics, respectively; (**C**) distribution of peptide numbers for quantified proteins; (**D**) numbers of identified p-sites and phosphoproteins; (**E**) distribution of quantified pS, pT, and pY residues; (**F**) the classification of p-sites based on LP scores; (**G**) comparison of identified p-sites with known p-sites curated from five public databases; and the PCA result of tumor and NAT samples from (**H**) proteomic and (**I**) phosphoproteomic data.

From the proteomic and phosphoproteomic profiling of CCAs, we identified 48,796 peptides and 13,017 phosphopeptides, respectively (Figure 2A and Table S3). From the distribution of spectral counts for identified peptides, there were 19,888 (40.76%) peptides and 6052 (46.49%) phosphopeptides that could be matched with ≥2 spectral counts (Figure 2B). Also, the average spectral counts were calculated as 1.83 and 2.45 for peptides and phosphopeptides, respectively. By mapping peptides to protein sequences of reference proteome, we obtained 7667 proteins, and only 14.88% (1141) of these proteins were identified with just one matched peptide, with an average number of 8.00 quantified peptides per protein (Figure 2C). From the phosphoproteomic data of CCA samples, we quantified 12,027 p-sites in 4088 phosphoproteins, including 10,393 phospho-serine (pS, 86.41%), 1508 phospho-threonine (pT, 12.54%), and 126 phospho-tyrosine (pY, 1.05%) residues (Figure 2D,E, and Table S3). Based on the localization probability (LP) scores of p-sites calculated from MaxQuant [21] and the previously reported classification rule [30], these p-sites were classified into 10,068 class I (LP > 0.75, 83.71%), 1635 class II (0.5 < LP ≤ 0.75, 13.59%), 323 class III (0.25 ≤ LP ≤ 0.5, 2.69%), and 1 class IV (LP < 0.25, 0.01%), respectively (Figure 2F). The majority of p-sites (11,623, 96.64%) quantified in this study were curated and annotated in at least one phosphorylation resource, by comparing with known p-sites of five public databases, including EPSD [23], dbPTM 2019 [24], PhosphoSitePlus [25], Phospho.ELM 9.0 [26], and UniProt [22] (Figure 2G). The intensity distribution of proteins, as well as the normalized intensity distribution of p-sites, were similar for different samples (Figure S1A,B). In addition, the PCA of proteomic and phosphoproteomic data were individually performed. The results exhibited that CCA tumors and their NATs could be distinguished in both protein and phosphorylation levels (Figure 2H,I).

### 3.3. The Landscapes of Protein Expression and Phosphorylation for CCAs

**Figure 3:**
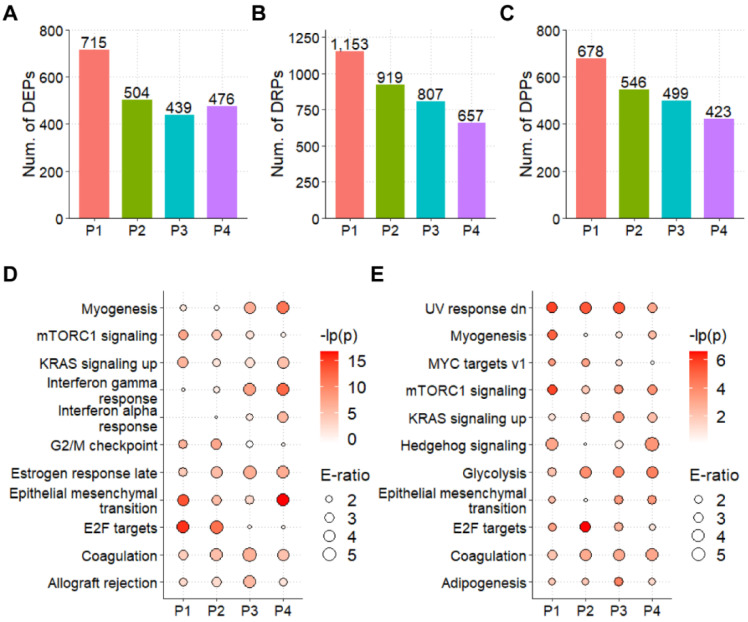
The functional enrichment analysis of CCAs: the numbers of (**A**) DEPs, (**B**) DPRs, and (**C**) DPPs of CCA tumors against their corresponding NATs; the MSigDB-based functional enrichment analysis for (**D**) DEPs and (**E**) DPPs.

From the proteomic profiling of tumors against corresponding NAT, we obtained 715, 504, 439, and 476 differentially expressed proteins (DEPs) with the two-fold change (≥2 or ≤0.5), respectively (Figure 3A). We detected 1153, 919, 807, and 657 DRPs from four patients, respectively (Figure 3B). At the level of phosphoproteins, there were 678, 546, 499, and 423 differentially phosphorylated proteins (DPPs) with at least one DRP (Figure 3C). Then, we performed the GO enrichment analysis of DEPs and DPPs (Figure S2). The results showed that DEPs mainly enriched in ribosome synthesis and cell cycle regulation in fields of biological process, while the DPPs mainly enriched in metabolic process. Also, the KEGG enrichment analysis was performed with the hallmark gene annotations from the MSigDB [27]. At the protein level of CCA, epithelial-mesenchymal transition (EMT), which is well characterized in CCA [31], was significantly up-regulated in all patients, as well as the coagulation process (Figure 3D). For the phosphorylated protein level of CCA, four hallmark processes, including glycolysis, coagulation, and mTORC1 signaling, were statistically up-regulated in all four patients (Figure 3E). The overlap of enriched processes between proteomic and phosphoproteomic data was minimal (Figure 3D,E) and suggested that distinct hallmark processes are regulated by protein or phosphorylation in CCA. Therefore, the proteomic and phosphoproteomic data could provide more information on the dynamic change of signaling in CCA.

### 3.4. The Integrated Pipeline for the Prediction of CCA-Associated PKs

**Figure 4:**
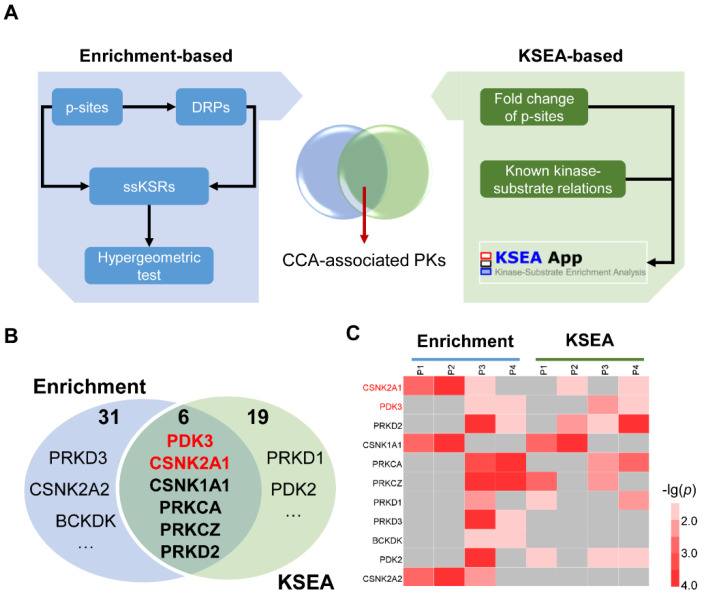
The prediction of CCA−associated PKs with CifPK pipeline: (**A**) the procedure of the CifPK pipeline, including the enrichment−based analysis, KSEA-based prediction, and the integration of PK candidates from two methods; (**B**) the Venn diagram of CCA−associated PKs predicted with enrichment− and KSEA−based method, with the known CCA−associated PKs shown in red; (**C**) the heatmap of differential dysregulation of 11 potentially CCA−associated PKs in CCA samples.

Theoretically, functional PKs in CCA could be measurable in tumors against their corresponding NATs. For example, the significantly changed phosphorylation level of the downstream substrates for one specific PK directly means this PK might be involved in regulating CCA. Therefore, we predicted the CCA-related PKs with the CifPK pipeline, which contains three steps: enrichment-based analysis, prediction with KSEA [20,28], and integration of predicted results (Figure 4A).

From the enrichment- and KSEA-based methods, 37 and 25 PKs, respectively, were predicted to associate with CCA (Figure 4B, Table S5). Moreover, the intersection of the two methods contained six potentially likely CCA-associated PKs, including CSNK2A1, PDK3, PRKD2, CSNK1A1, PRKCA, and PRKCZ (Figure 4B and Table S5). Specifically, two predicted PKs, CSNK2A1 [32] and PDK3 [33], were reported as functional regulators in CCA. In addition, based on the known functional roles in cancer and the reported classification of the kinase family [34], PRKD1, PRKD3, BCKDK, PDK2, and CSNK2A2 were considered as potential CCA-relative PKs, which were related to the six overlapped PKs (Figure 4B and Table S5). For the eleven candidate PKs, the corresponding p values were illustrated for the changes in PKs-based enrichment and KSEA methods (Figure 4C). Most of these PK proteins (9, 81.82%) were quantified in all samples without any one DEP (Figure S3A). Meanwhile, the p-sites of 4 potentially CCA-associated PKs were quantified, and none of these p-sites were significantly regulated in all four patients (Figure S3B). These results suggested that directly identifying CCA-associated PKs from proteomic and phosphoproteomic data was a great challenge.

### 3.5. Screening of PK Candidates

**Figure 5:**
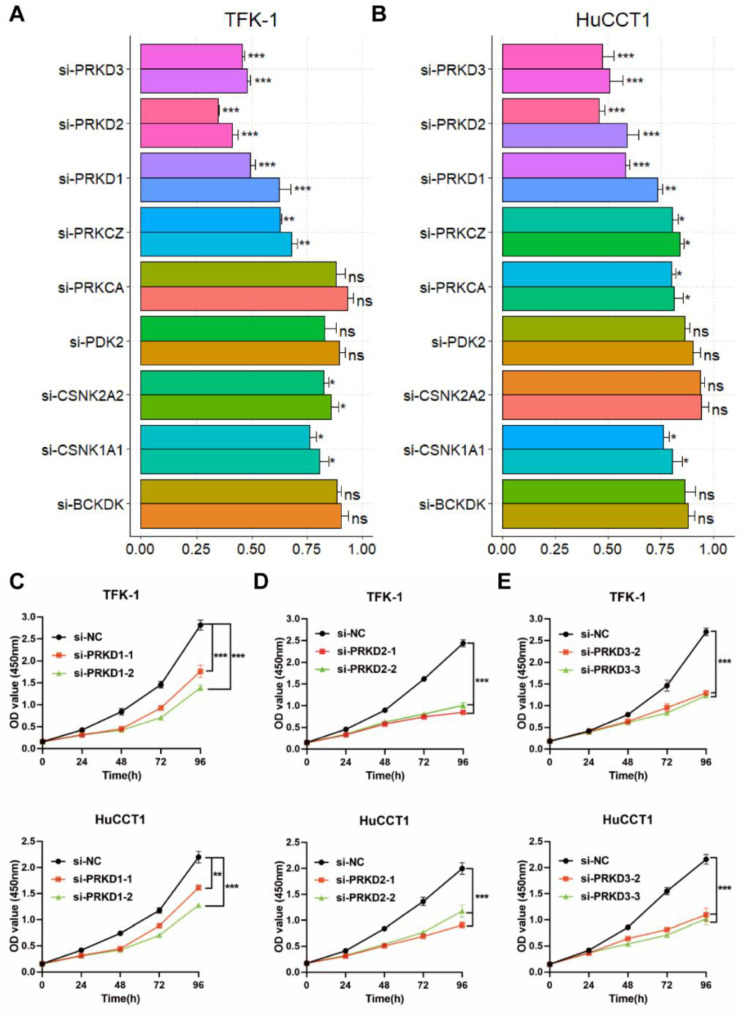
Screening PK candidates with siRNAs: Relative proliferation of (**A**) TFK-1 and (**B**) HuCCT1 cells, transfected siRNA for each PK candidate, were measured by CCK-8 assay. Proliferation of TFK-1 and HuCCT1 cells treated with siRNAs for (**C**) PRKD1, (**D**) PRKD2, or (**E**) PRKD3 was measured by using the CCK-8 assay. * *p* < 0.05, ** *p* < 0.01, *** *p* < 0.001, *p*-value was determined by two-way ANOVA. Data are presented with means ± SDs and from three independent experiments.

To confirm the prediction above, a WB assay was performed with CCA tissues and cell lines. The results suggested that PRKD family members and the critical corresponding substrates, phospho-Src-S17 and phospho-β-catenin-S552, were expressed in both CCA tissues and cell lines (Figure S4). Then, in order to investigate whether the kinases resulting from the analysis above play a role in CCA, we further validated the effect of nine candidate PKs on CCA proliferation. After silencing the expression of each candidate PK with siRNA (Figure S3 and Table S4), the CCK8 assay was performed in TFK-1 and HuCCT1, respectively. The results suggested that five of the nine candidate PKs, including PRKD1, PRKD2, PRKD3, PRKCZ, and CSNK1A1, showed potently suppressed effects on the cell proliferation in both TFK-1 and HuCCT1 (Figure 5A,B). The detail of the inhibition effects in cell proliferation for other PKs were shown in Figure 5C–E and Figure S5. Interestingly, the top three PKs, PRKD1, PRKD2, and PRKD3, which belong to the protein kinase D (PKD) family of the calcium/calmodulin-dependent kinase (CAMK) group, showed a consistently strong effect on the proliferation inhibition of CCA cells (Figure 5C–E).

### 3.6. The Inhibition of PKDs Suppressed the Migration and Invasion of CCA

**Figure 6:**
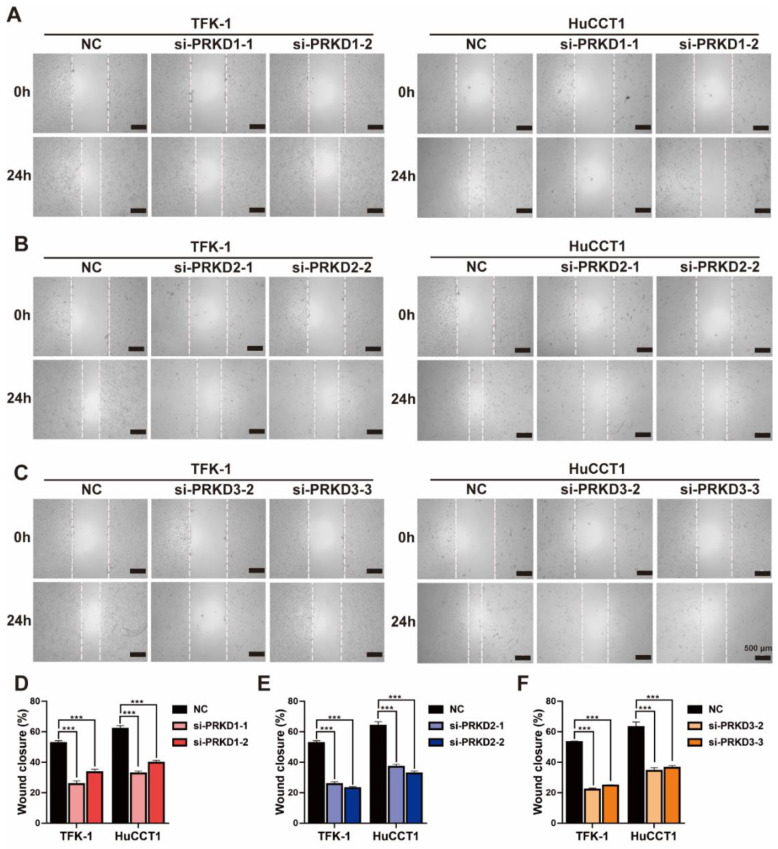
The wound healing assays of CCA cells treated with PKD specific siRNAs: representative phase-contrast images of TFK-1 and HuCCT1 cells at 0 and 24 h transfection with effective siRNAs for (**A**) PRKD1, (**B**) PRKD2, and (**C**) PRKD3, respectively (scale bars = 500 μm); percentage of wound closure that occurred at 24 h after the transfection of each specific siRNA for (**D**) PRKD1, (**E**) PRKD2, and (**F**) PRKD3, respectively. *** *p* < 0.001, determined by two-way ANOVA. Data are presented with means ± SDs and from three independent experiments.

**Figure 7:**
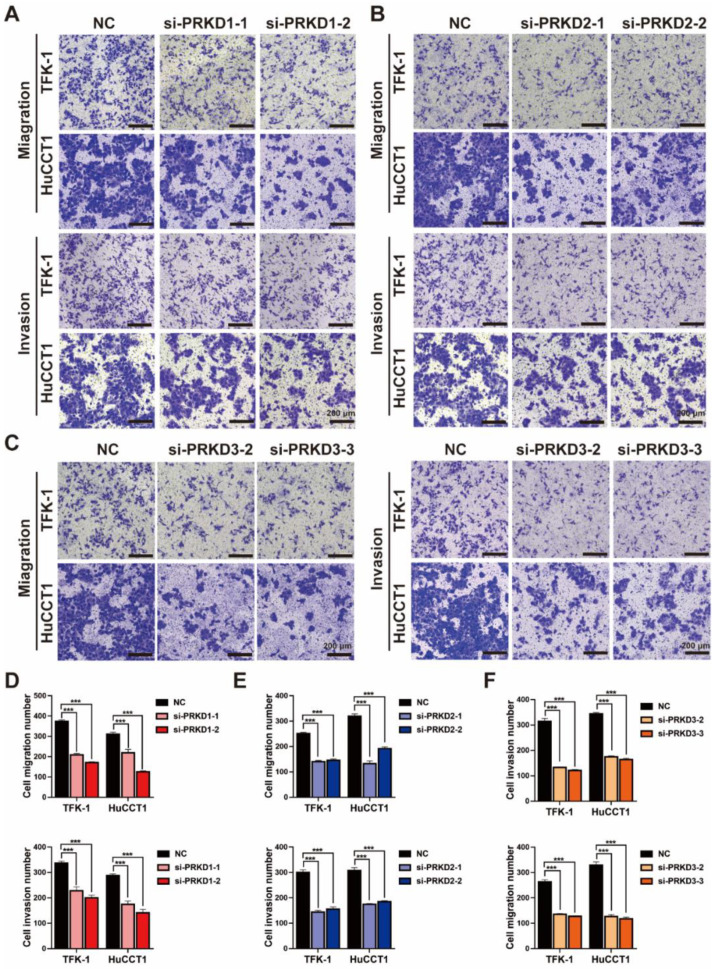
Experimental analysis of functional roles for PKD family in CCA cells: Transwell assay of TFK-1 and HuCCT1 treated with each specific siRNA for (**A**) PRKD1, (**B**) PRKD2, and (**C**) PRKD3, respectively (scale bars = 200 μm); the number of migrated and invaded cells for (**D**) PRKD1, (**E**) PRKD2, and (**F**) PRKD3, respectively. *** *p* < 0.001 determined by two-way ANOVA. Data are presented with means ± SDs and from three independent experiments.

To further investigate the role of PKD family genes in CCA, we performed the wound healing assay in both TFK-1 and HuCCT1, treated with each siRNA for PKs of the PKD family (Figure 6A–C) and found that all siRNAs of PKD family members reduced 50% of wound closure (Figure 6D–F). Also, the Transwell assay showed that silencing PRKD1/2/3 expression could significantly suppress CCA′s migration and invasion capacity (Figure 7).

**Figure 8:**
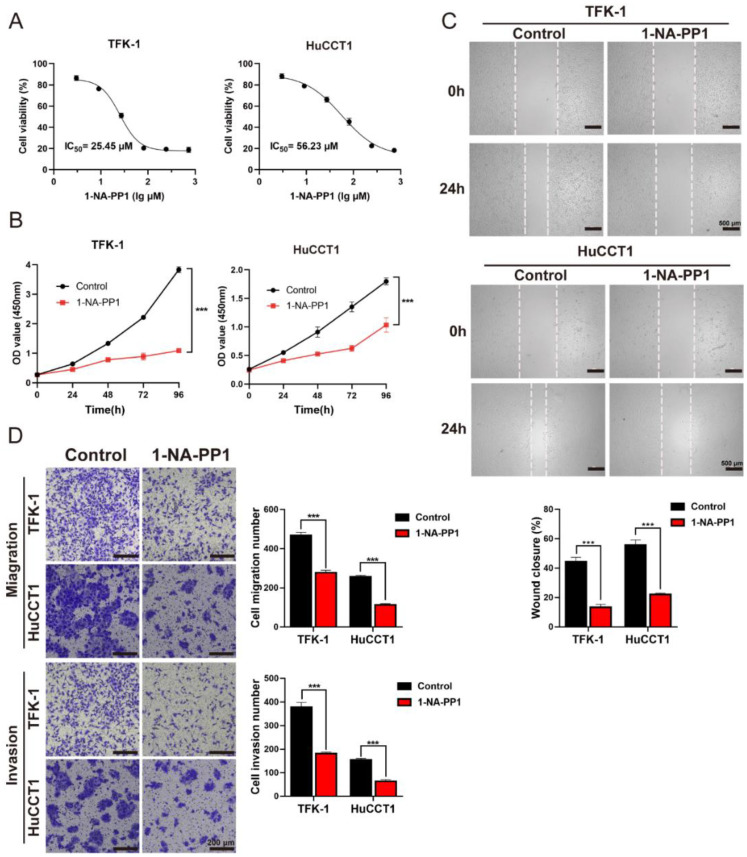
Experimental analysis of 1-NA-PP1 in CCA cells: (**A**) CCK8 assay was performed to measure the median inhibition concentration (IC_50_) on TFK-1 and HuCCT1 cells; (**B**) proliferation of TFK-1 and HuCCT1 cells treated with 1-NA-PP1 was measured by CCK-8 assays; (**C**) representative phase-contrast images of TFK-1 (top) and HuCCT1 (bottom) cells at 0 and 24 h treatment with 1-NA-PP1. Scale bars = 500 μm. (**D**) The Transwell migration and invasion assays of TFK-1 and HuCCT1 cells were after the 24 h treatment with 1-NA-PP1; the concentration of 1-NA-PP1 in treating TFK-1 and HuCCT1 was 25.45 μm and 56.23 μm, respectively. Scale bars = 200 μm. *** *p* < 0.001, determined by two-way ANOVA. Data are presented with means ± SDs and from three independent experiments.

To interrogate whether inhibition of the kinase activity of the PKD family would suppress the cell proliferation and migration of CCA cells, 1-naphthyl PP1 (1-NA-PP1), which is a selective and effective pan-inhibitor of the PKD family [35], was used to treat both TFK-1 and HuCCT1 cells (Figure 8). The IC_50_ values of 1-NA-PP1 were 25.45 μm and 56.23 μm for TFK-1 and HuCCT1 cells, respectively (Figure 8A). The inhibition of the PKD family by 1-NA-PP1 showed a substantial decrease in cell proliferation of TFK-1 cells and a weak decrease in HuCCT1 cells against negative control (Figure 8B). Furthermore, the wound healing assays showed that 1-NA-PP1 significantly inhibited 50% of wound closure (Figure 8C). In addition, 1-NA-PP1 markedly suppressed migration and invasion of TFK-1 and HuCCT1, with a more than 50% decrease (Figure 8D). Taken together, the inhibition of PK members of the PKD family by either siRNA or the inhibitor significantly suppressed the proliferation, migration, and invasion ability of CCA cells.

## 4. Discussion

CCA is one of the most malignant and aggressive adenocarcinomas, and the majority of CCAs lack effective drug therapies [1,2]. Recent advances in the highly detailed systematic quantifications by proteomics and phosphoproteomics have provided an excellent opportunity to acquire the molecular signaling of protein expression and phosphorylation modifications in tumors. In this study, the TMT-labeling phosphoproteomics was performed in four poorly-differentiated CCA tissues and NATs; we quantified 12,027 p-sites in 4088 phosphoproteins. According to the distribution of DRPs, most phosphorylation modification sites were located in serine and threonine, which was consistent with previous reports [36]. This suggested that serine- and threonine-phospho-sites should be the focus of phosphorylation studies in CCA. The motif enrichment heatmap showed a significant preference for negatively charged glutamate (E) and aspartate (D) around the modified serine and threonine residues (Figure S6), which may help understand the preference of enzymes for their substrates. Further, the functional enrichment analysis suggested a highly activated biosynthesis, metabolism, and EMT state in CCA, which was in accordance with malignant tumors characterized by increased proliferation and metastatic capacity.

Up to the present time, some researchers have applied phosphoproteomics to investigate dysregulated phosphorylation events. In 2017, Qian et al. found that the phosphorylation of serine 59 residue in specificity protein 1 (Sp1) was up-regulated by atypical protein kinase C iota (PRKCI) in CCA through the phosphoproteomic data based on the labeling of isobaric tags for relative and absolute quantitation (iTRAQ) [37]. Khorsandi et al. performed label-free phosphoproteomic quantifications of thirteen CCA tumors with corresponding normal adjacent tissues (NATs) and seven CCA cell lines; the KSEA-based analysis suggested CDK and MAPK as potential CCA-related PKs, which were also suggested in the result of KSEA-based prediction in this study (Table S4). Moreover, the inhibitors of the PI3K/ATK/mTOR pathway were proved to suppress the proliferation of CCA cells [16], which were similarly up-regulated in this work. Dong et al. performed proteomic profiling of intrahepatic cholangiocarcinoma (iCCA) with paired tumors and adjacent liver tissue from 262 patients; the results suggested that FGFR fusion and KRAS mutation were significant and crucial for iCCA development, and that they significantly activated MAPK, mTOR, and Rho GTPase signaling pathways [38]. FGFR alterations are known as oncogenic drivers in CCA; while it is only evident in iCCA with an approximate incidence of 20%, it is not observed in extrahepatic cholangiocarcinoma (eCCA) and NATs [17]. Although FGFR was not identified in this study, its binding protein, the phospho-GRB2-S90, was significantly down-regulated in CCA, which showed agreement with the report above.

The upstream PKs of dysregulated phosphorylation events are involved in CCA′s development and should be considered as potential drug targets. Due to the importance and complexity of PK-mediated regulations, the systematic evaluation of functionally central PKs regulating CCA is fundamental for the clinical diagnosis and therapeutics for CCA. In this work, we obtained the molecular landscapes of protein expression and phosphorylation modifications for CCA based on the proteomic and phosphoproteomic profiling of poorly differentiated CCA tumors with corresponding NATs. Then, we designed a new pipeline named CifPK, which inferred eleven highly potential PKs associated with CCA and experimentally validated five newly CCA-associated PKs. All the validated CCA-associated PKs identified in this study showed no expression difference between cancer and NATs, which suggested that it would be difficult to obtain valid information directly from mass spectrometry data, further highlighting the importance of bioinformatics-based approaches for protein kinase prediction. The genes of the PKD family encode three serine/threonine protein kinases, which are conserved in mammals, including PRKD1, PRKD2, and PRKD3, also known as PKD1, PKD2, and PKD3 [39]. Previously, the PKD family was reported to act as a promoter in glioblastoma [40], gastric cancer [41], prostate cancer, and breast cancer [42]. Consistently, the PKD kinase family members, including PRKD1, PRKD2, and PRKD3, were identified as putative oncogenes in this study, and its inhibitor could be considered a potentially novel treatment for CCA.

Indeed, the sample size included in this study was insufficient; more samples and work are needed to prove the role of PRKD kinases in CCA development, including in vivo assay, prognostic analysis in numerous clinical samples, and molecular mechanisms. However, we identified 4088 phospho-proteins with 12,027 phospho-sites in CCA and successfully predicted and validated five CCA-related PKs in this study. Among them, PRKD family members served a crucial role in CCA development, and the PRKD inhibitor, 1-NA-PP1, could significantly suppress malignant biological behavior in CCA. Our study may provide a new perspective and potential targets for the treatment of CCA.

## Data Availability

The mass spectrometry proteomics data in this study have been deposited in the ProteomeXchange Consortium via the PRoteomics IDEntifications (PRIDE) [43] partner repository with the dataset identifier PXD035652 (http://www.ebi.ac.uk/pride) (accessed on 1 August 2022).

## Supplementary Materials

The following supporting information can be downloaded at: https://www.mdpi.com/article/10.3390/cells11193088/s1, Figure S1: Additional analysis of proteomic and phosphoproteomic profiling for CCAs. Figure S2: Gene Ontology (GO) enrichment analysis of the DEPs and DPPs. Figure S3: The protein abundance and phosphorylation modification of potentially CCA-associated PKs from proteomic and phosphoproteomic data. Figure S4: Western blot assay validated the expression of the candidate PKs and substrates in CCA tissues and cell lines. Figure S5: The CCK-8 assay of PK candidates. Figure S6: The heatmap of the amino acid profile of the serine-phosphosites and threonine-phosphosites. Table S1: Characteristics for patients. Table S2: TMT labeling data. Table S3: The proteomic and phosphoproteomic profiling of CCAs. Table S4: List of siRNA. Table S5: List of predicted kinase from different methods.

## Abbreviations

BRAF	Serine/threonine-protein kinase B-raf
CCA	Cholangiocarcinoma
CifPK	Comprehensive inference of functional protein kinases
CCK-8	Cell Counting Kit-8
DEPs	Differentially expressed proteins
DRPs	Differentially regulated p-sites
DPPs	Differentially phosphorylated proteins
EMT	Epithelial mesenchymal transition
eCCA	Extrahepatic cholangiocarcinoma
FGFR2	Fibroblast growth factor receptor 2
FDA	Food and Drug Administration
HCC	Hepatocellular carcinoma
IC_50_	Half-maximal inhibitory concentration
iCCA	Intrahepatic cholangiocarcinoma
LC-MS/MS	Liquid chromatography with tandem mass spectrometry
mTOR	Mammalian target of rapamycin
NATs	Normal adjacent tissues
PKs	Protein kinases
PKD/PRKD	Protein kinase D
PI3K	Phosphoinositide 3-kinase
PRKCI	Atypical protein kinase C iota
RTK	Receptor tyrosine kinase
Sp1	Specificity protein 1
siRNAs	Small interfering RNAs
1-NA-PP1	1-naphthyl PP1
