# Supplementary material for: Proteomic and Phosphoproteomic Profiling Reveals the Oncogenic Role of Protein Kinase D Family Kinases in Cholangiocarcinoma
Source: Cells. 2022 Sep 30;11(19):3088. doi: 10.3390/cells11193088 (PMC9562908; doi:10.3390/cells11193088)
Supplement: Supplementary file 1 [file cells-11-03088-s001.zip › cells-1871545-supplementary/Cells-1871545-supplementary-proofreading.pdf]

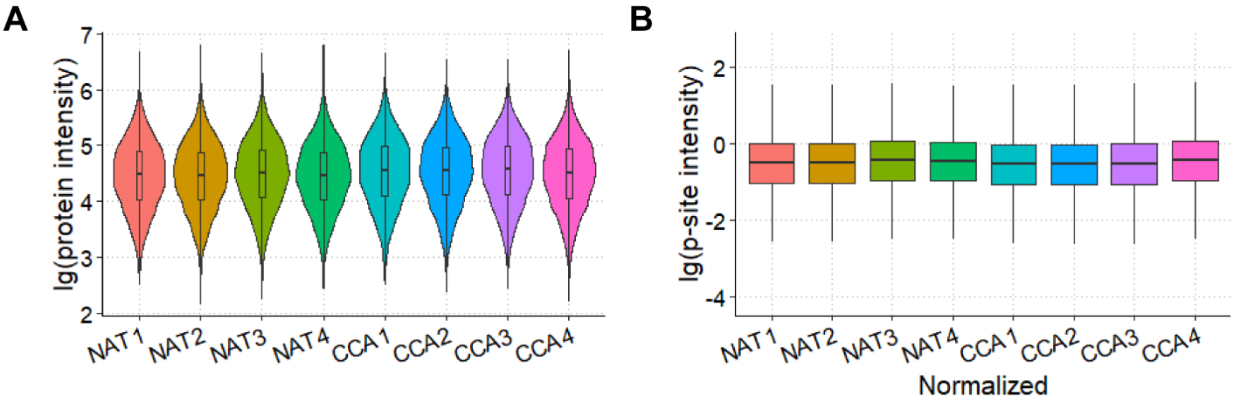

Figure S1. Additional analysis of proteomic and phosphoproteomic profiling for CCAs.
A, B. The intensity distribution of quantified proteins (A) and normalized p-sites (B) in all CCA patients.

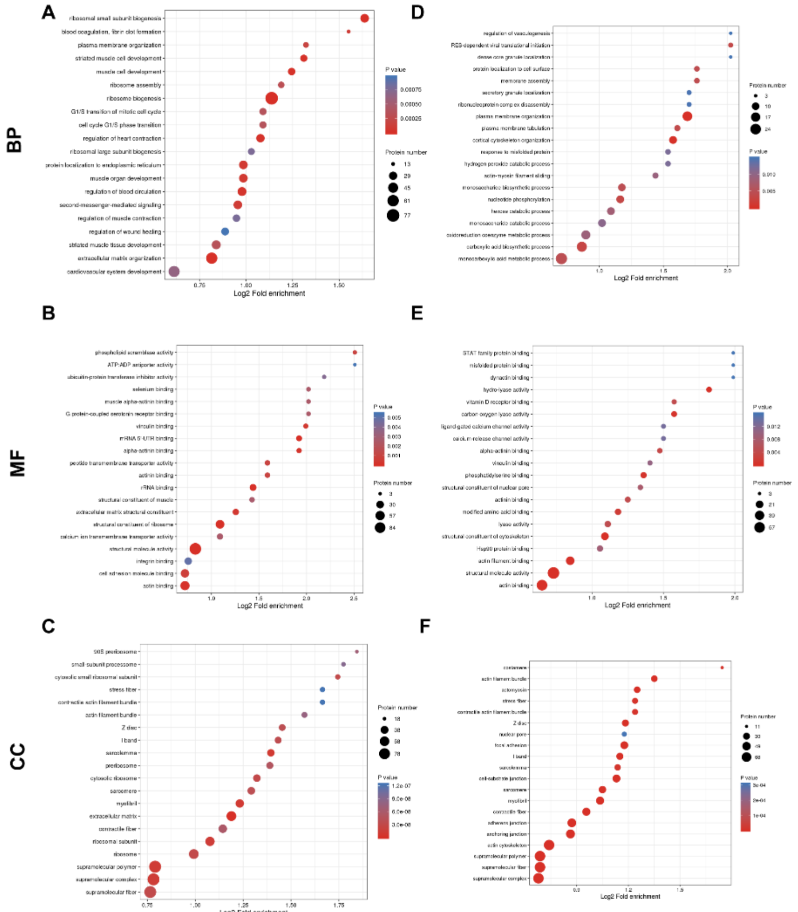

Figure S2. Gene Ontology (GO) enrichment analysis of the DEPs **(A-C)** and DPPs **(D-F)** based on the biological process (BP), molecular function (MF), and cellular component (CC).

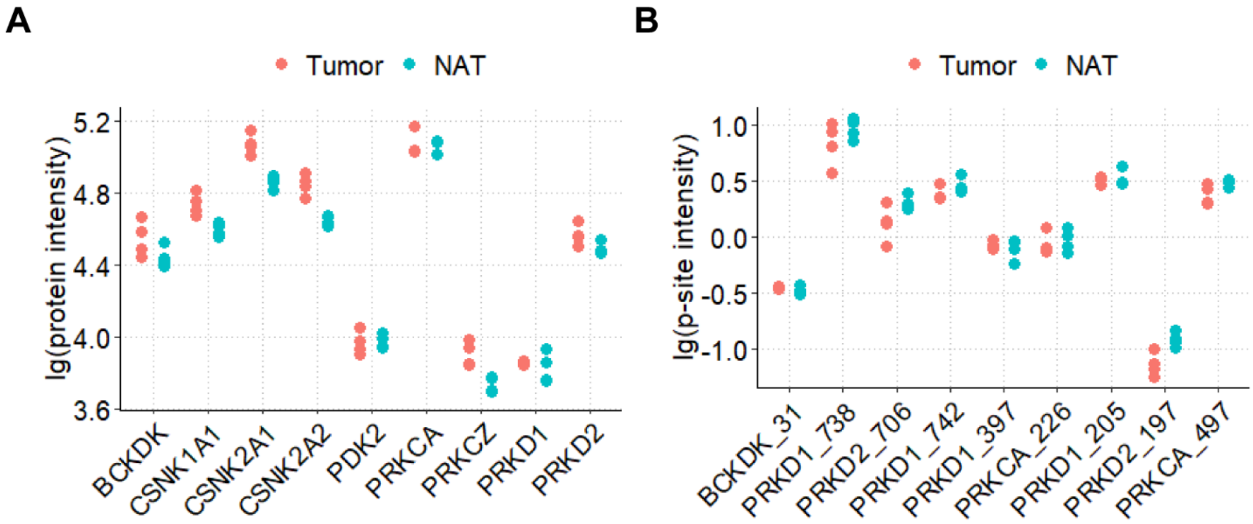

Figure S3. The protein abundance and phosphorylation modification of potentially CCA-associated PKs from proteomic and phosphoproteomic data. A. The protein abundance of 11 potentially CCA-associated PKs from proteomic data, if available. **B.** The phosphorylation modification of potentially CCA-associated PKs from phosphoproteomic data, if available.

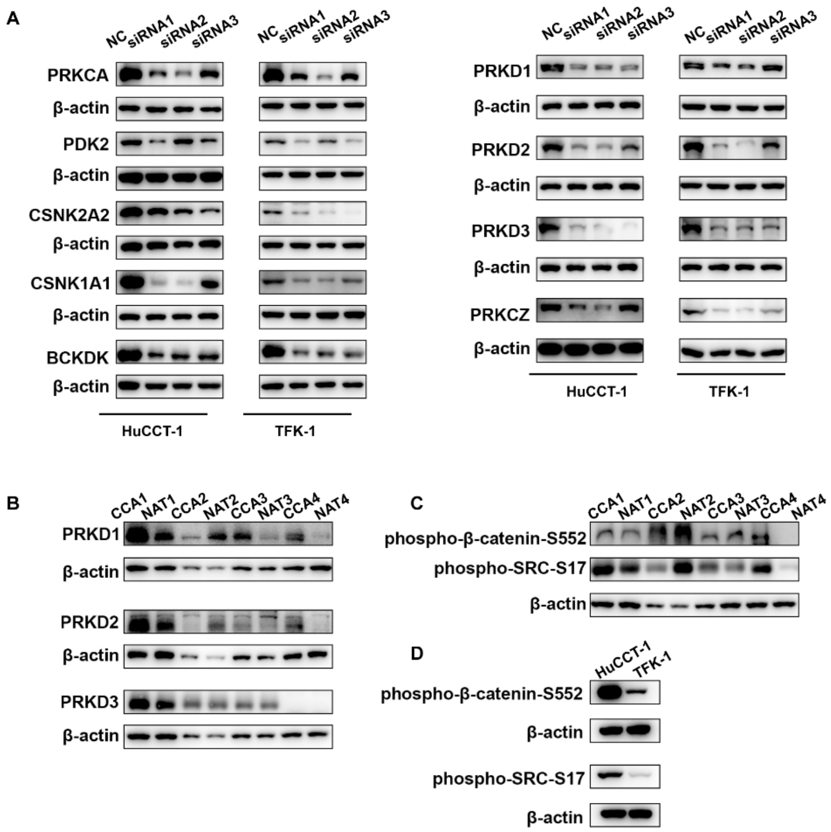

Figure S4. Western blot assay validated the expression of the candidate PKs and substrates in CCA tissues and cell lines. **A.** Western blot for measuring the expression of PK treated with siRNA for 48 h, including PRKCA, PDK2, CSNK2A2, CSNK1A1,BCKDK, PRKD1, PRKD2, PRKD3, and PRKCZ. **B.** The expression of PRKD1, PRKD2, and PRKD3 in CCA tissues and pared NATs was measured by western blot. **C.** The expression of PRKD-relative substrates in CCA tissues and pared NATs was validated by western blot, including phospho-β-catenin-S552 and phospho-Src-S17. **D**. The expression of phospho-β-catenin-S552 and phospho-Src-S17 in TFK-1 and HuCCT1 were measured by western blot assay.

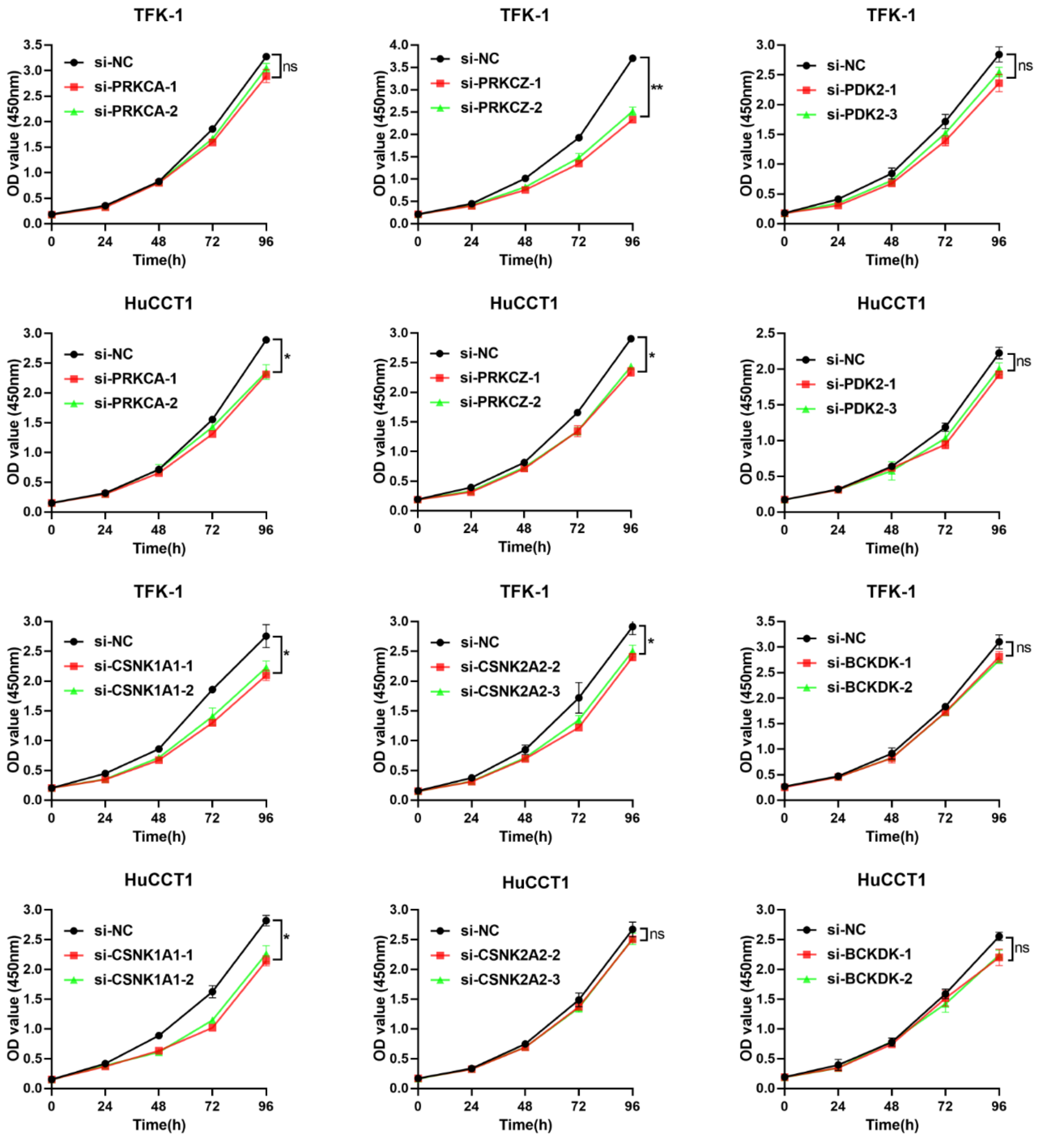

.
Figure S5. The CCK-8 assay of PK candidates. **A-F**. The proliferation of TFK-1 and HuCCT1 transfected with specific siRNAs for PK candidates, including PRKCA (**A**), PRKCZ (**B**), PDK2 (**C**), CSNK1A1 (**D**), CSNK2A2 (**E**), and BCKDK (**F**). $^*p < 0.05$, $^{**}p < 0.01$, $^{***}p < 0.001$, determined by two-way ANOVA. Data are presented with means ± SDs and from three independent experiments.

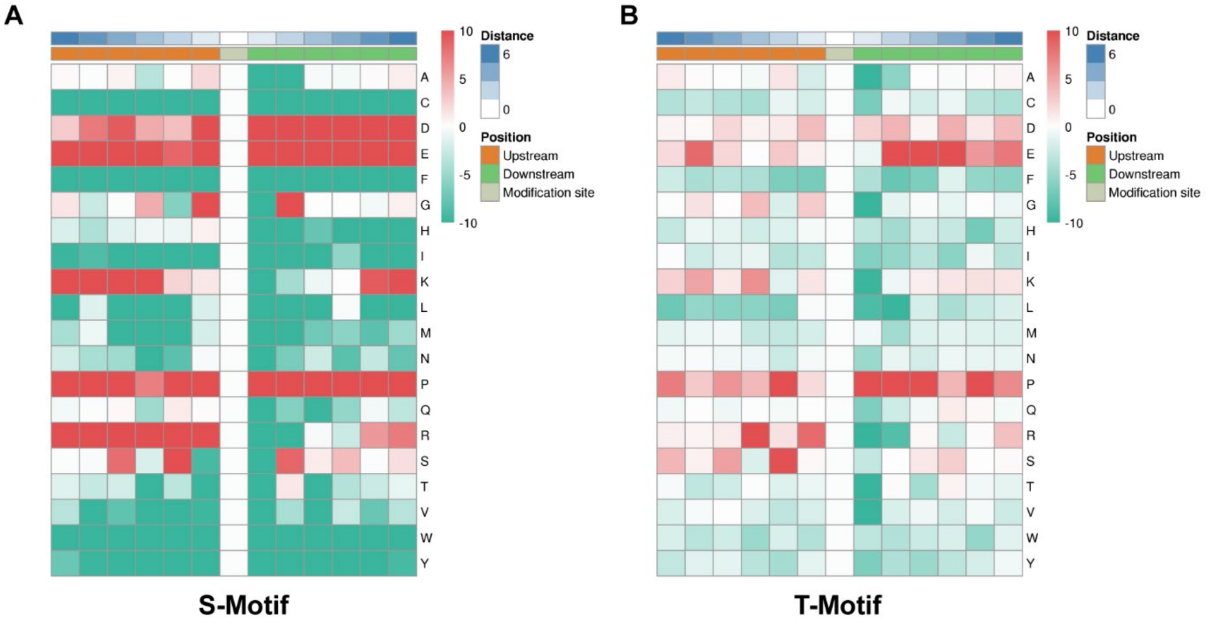

Figure S6. The heatmap of the amino acid profile of the serine-phosphosites **(A)** and threonine-phosphosites **(B)**, featuring the enrichment (red) and depletion (green) of the amino acids at every position (from -10 to +10) on both sides of the phospho-site.
