# Supplementary material for: Proteomic and Phosphoproteomic Profiling Reveals the Oncogenic Role of Protein Kinase D Family Kinases in Cholangiocarcinoma
Source: Cells. 2022 Sep 30;11(19):3088. doi: 10.3390/cells11193088 (PMC9562908; doi:10.3390/cells11193088)
Supplement: Supplementary file 1 [file cells-11-03088-s001.zip › cells-1871545-supplementary/Table S2 TMT labeling data.pdf]

The labeling efficiency test in this study showed that the total number of peptides detected in the single shot was 5282 and the number of N-terminal labeled peptides was 5221, with a labeling efficiency text of 98.85%.

Marking efficiency refers to the results of the whole group of samples, each sample is mixed in equal amounts for marking efficiency testing, so there is no marking efficiency data for a single sample; however, the intensity values of the samples can be used to reflect the marking efficiency of individual samples, the intensity values of individual samples for this project all fluctuate within ±5%, indicating that the marking efficiency of individual samples is basically the same, the specific intensity data are as follows.

Sample	a137Ca	a138P	a145Ca	a146P	a421Ca	a422P	a443Ca	a444P
Label	127N	127C	128N	128C	129N	129C	130N	130C
Raw signal strength	1112282	1092575	1156127	1092115	1155070	1183981	1132193	1111954
Normalised intensity values	0.98	0.97	1.02	0.97	1.02	1.05	1.00	0.98
